# Complete mitochondrial genome of *Zeugodacus tau* (Insecta: Tephritidae) and differentiation of *Z*. *tau* species complex by mitochondrial cytochrome *c* oxidase subunit I gene

**DOI:** 10.1371/journal.pone.0189325

**Published:** 2017-12-07

**Authors:** Hoi-Sen Yong, Sze-Looi Song, Phaik-Eem Lim, Praphathip Eamsobhana

**Affiliations:** 1 Institute of Biological Sciences, Faculty of Science, University of Malaya, Kuala Lumpur, Malaysia; 2 Institute of Ocean and Earth Sciences, University of Malaya, Kuala Lumpur, Malaysia; 3 Department of Parasitology, Faculty of Medicine Siriraj Hospital, Mahidol University, Bangkok, Thailand; SOUTHWEST UNIVERSITY, CHINA

## Abstract

The tephritid fruit fly *Zeugodacus tau* (Walker) is a polyphagous fruit pest of economic importance in Asia. Studies based on genetic markers indicate that it forms a species complex. We report here (1) the complete mitogenome of *Z*. *tau* from Malaysia and comparison with that of China as well as the mitogenome of other congeners, and (2) the relationship of *Z*. *tau* taxa from different geographical regions based on sequences of cytochrome *c* oxidase subunit I gene. The complete mitogenome of *Z*. *tau* had a total length of 15631 bp for the Malaysian specimen (ZT3) and 15835 bp for the China specimen (ZT1), with similar gene order comprising 37 genes (13 protein-coding genes—PCGs, 2 rRNA genes, and 22 tRNA genes) and a non-coding A + T-rich control region (D-loop). Based on 13 PCGs and 15 mt-genes, *Z*. *tau* NC_027290 (China) and *Z*. *tau* ZT1 (China) formed a sister group in the lineage containing also *Z*. *tau* ZT3 (Malaysia). Phylogenetic analysis based on partial sequences of *cox1* gene indicates that the taxa from China, Japan, Laos, Malaysia, Bangladesh, India, Sri Lanka, and *Z*. *tau* sp. A from Thailand belong to *Z*. *tau* sensu stricto. A complete *cox1* gene (or 13 PCGs or 15 mt-genes) instead of partial sequence is more appropriate for determining phylogenetic relationship.

## Introduction

*Zeugodacus tau* (Walker) is the most common tephritid fruit fly species of the genus *Zeugodacus* found in Southeast Asia [1]. It is among the economically important species belonging to the Dacinae subfamily, occurring from Pakistan to Philippines and south to Indonesia [2]. It is a polyphagous fruit pest, infesting host fruits of the families Anacardiaceae, Cucurbitaceae, Elaeocarpaceae, Moraceae, Myrtaceae, Oxalidaceae, Rutaceae, Sapotaceae, and Solanaceae [3–7]. The adult male flies are attracted to Cue lure.

Studies based on cytogenetics, partial sequences of mitochondrial cytochrome *c* oxidase subunit I (*cox1*) gene and allozymes have revealed that *Z*. *tau* (previously referred to as *Bactrocera tau* (Walker)) is a species complex comprising eight species (or morphs) in Thailand, with species A designated as *Z*. *tau* sensu stricto [8–10]. *Z*. *tau* A may be reliably separated from *Z*. *tau* B, C, D, E, F, G, and I by the heat shock protein 70 cognate gene *Bthsc*1 [11].

Phylogenetic analysis using mitochondrial *cox1* gene sequences revealed that the *Z*. *tau* population in Himachal Pradesh (India) is closely related to *Z*. *tau* sp. A from Thailand [12]. The overall genetic variability in this Indian taxon is substantial, with 10 different haplotypes detected in 16 individuals. A study of 23 *Z*. *tau* populations (Myanmar and western Yunnan; Laos and southern Yunnan; central Yunnan; Thailand; southern China, central China and northern Vietnam; and southwestern China), based on mitochondrial NADH dehydrogenase gene (*nad1*), revealed six genetic groups corresponding to geographical characteristics, and strong genetic structure for the populations in western China, Thailand, and Laos [13]. *Z*. *tau* in China has also been reported to exhibit seven cytochrome *b* haplotypes (NCBI GenBank 26-JUL-2016: AY953491-AY953497).

To date, there is only a single report on the complete mitochondrial genome (mitogenome) of *Z*. *tau* [14]. The taxon is from Shenzhen, China. We report here the complete mitogenome of *Z*. *tau* from Malaysia and compare it to that of China as well as the mitogenome of other congeners. We also carry out phylogenetic analysis using *cox1* gene to determine the relationship of *Z*. *tau* taxa from different geographical regions.

## Materials and methods

### Specimen collection and mitochondrial DNA extraction

Male fruit flies of *Z*. *tau* were collected in Malaysia (Kuala Lumpur– 3.1390°N, 101.6869°E) and China (Zhuhai, Guangdong– 22.2710°N, 113.5767°E) by means of Cue lure according to the method of Yong *et al*. [15]. The specimens were preserved in absolute ethanol and stored in -20°C freezer until use. *Z*. *tau* is an insect pest. It is not endangered or protected by law. No permits are required to study this fruit fly. The extraction of mitochondrial DNA was according to the method of Yong *et al*. [16].

### Library preparation, genome sequencing and analysis

Sample and library preparation (using Nextera DNA Sample Preparation Kit), genome sequencing using the Illumina MiSeq Desktop Sequencer (2 × 150 bp paired-end reads) (Illumina, USA), and genome analysis were as described in Yong *et al*. [15–16]. The mitogenome sequences have been deposited in GenBank–accession number MF966383 (ZT1) and MF966384 (ZT3).

### Mitogenomes and cytochrome *c* oxidase subunit I sequences from GenBank

The complete mitogenomes of Tephritidae available from GenBank (Table 1) were used for phylogenetic comparison. Species of *Drosophila*–*D*. *incompta* Wheeler & Takada NC_025936 [17]; *D*. *melanogaster* Meigen NC_024511 (unpublished); and *D*. *yakuba* Burla NC_001322 [18]–were used as outgroup taxa. Representative *cox1* sequences of *Z*. *tau* from different geographic regions were used for reconstruction of phylogenetic tree.

**Table 1:** Complete mitogenomes of Tephritidae available from GenBank.

Taxon	Accession no	Reference
*Bactrocera arecae* (Hardy & Adachi)	NC_028327	[15]
*Bactrocera carambolae Drew* & Hancock	NC_009772	Unpublished
*Bactrocera correcta* (Bezzi)	NC_018787	Unpublished
*Bactrocera dorsalis* (Hendel)	NC_008748	Unpublished
*Bactrocera dorsalis* (Hendel) (= *papayae* Drew & Hancock)	NC_009770	Unpublished
*Bactrocera dorsalis* (Hendel) (= *philippinensis* Drew & Hancock)	NC_009771	Unpublished
*Bactrocera dorsalis* (Hendel) (= *invadens* Drew, Tsura & White)	NC_031388	[19]
*Bactrocera latifrons* (Hendel)	NC_029466	[20]
*Bactrocera melastomatos* Drew & Hancock	NC_029467	[20]
*Bactrocera ritsemai* (Weyenbergh)	MF668132	Unpublished
*Bactrocera tryoni* (Froggatt)	NC_014611	[21]
*Bactrocera umbrosa* (Fabricius)	NC_029468	[20]
*Bactrocera zonata* (Saunders)	NC_027725	[22]
*Bactrocera (Daculus) oleae* (Rossi)	NC_005333	[23]
*Bactrocera (Tetradacus) minax* (Enderlein)	NC_014402	Unpublished
*Zeugodacus caudatus* (Fabricius) Malaysia	KT625491	[24]
*Zeugodacus caudatus* (Fabricius) Indonesia	KT625492	[24]
*Zeugodacus cucurbitae* (Coquillett)	NC_027254	Unpublished
*Zeugodacus depressus* Shiraki	KY131831	[25]
*Zeugodacus diaphorus* (Hendel)	NC_028347	[26]
*Zeugodacus scutellatus* (Hendel)	NC_027254	Unpublished
*Zeugodacus tau* (Walker)	NC_027290	[14]
*Ceratitis capitata* (Wiedemann)	NC_000857	[27]
*Ceratitis fasciventris* (Bezzi)	KY436396	[28]
*Dacus longicornis* Wiedemann	NC_032690	[29]
*Anastrepha fraterculus* (Wiedemann)	NC_034912	[30]
*Procecidochares utilis* Stone	NC_020463	Unpublished

### Phylogenetic analysis

Alignment of nucleotide sequences and reconstruction of phylograms based on 15 mt-genes and cytochrome *c* oxidase subunit I gene sequences followed that described in Yong *et al*. [26].

## Results

### Mitogenome features

The raw/final sequencing reads produced by next-generation sequencing on Illumina MiSeq Sequencer were 3286014/3205571 for *Z*. *tau* ZT3 (Malaysia) and 3191750/3113181 for *Z*. *tau* ZT1 (China).

**Fig 1:**
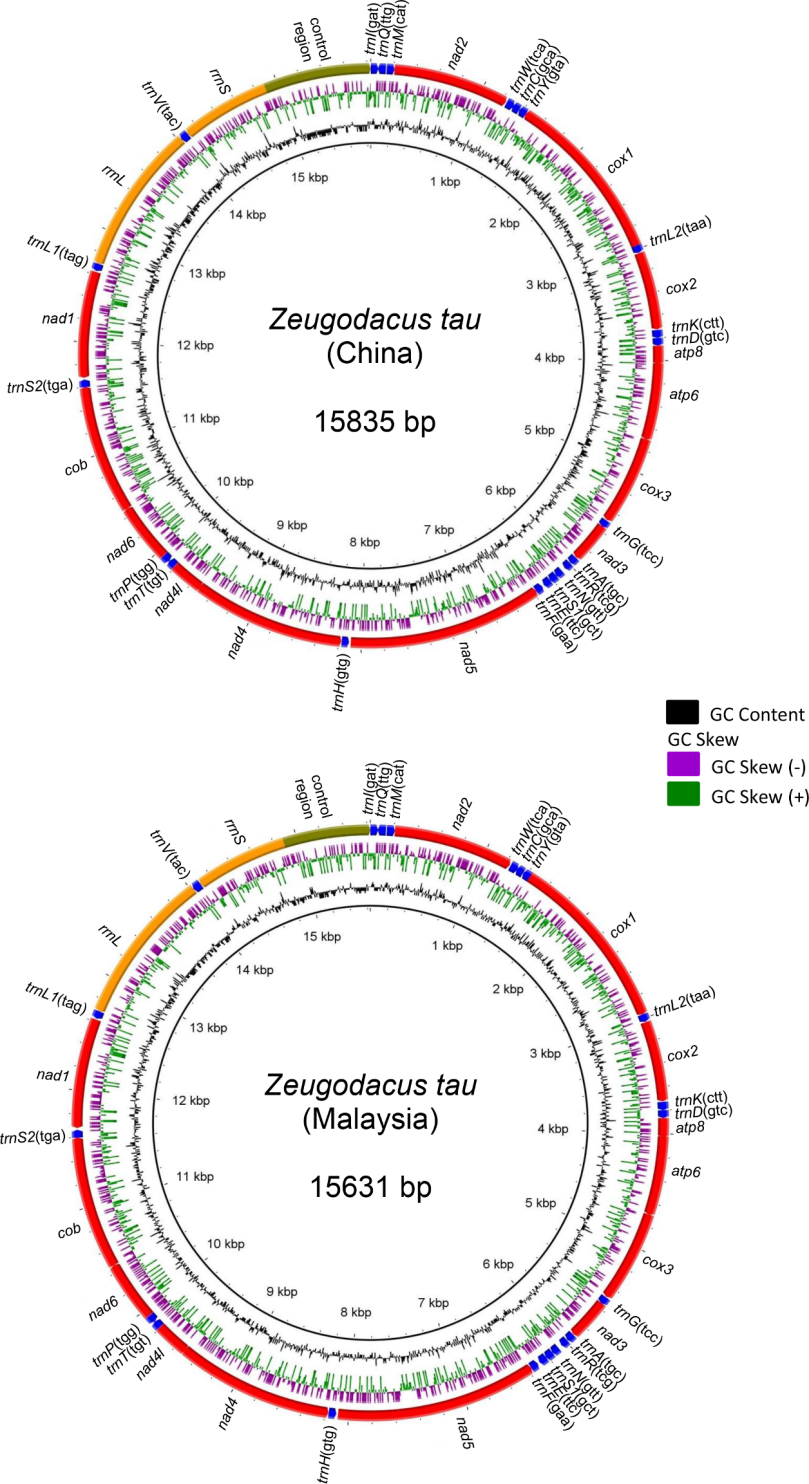
Complete mitogenomes of *Zeugodacus tau* ZT3 and *Z*. *tau* ZT1 with BRIG visualization showing the protein-coding genes, rRNA and tRNA genes. GC skew is shown on the outer surface of the ring whereas GC content is shown on the inner surface. The anticodon of each tRNAs is shown in bracket.

The complete mitogenome of *Z*. *tau* had a total length of 15631 bp for the Malaysian specimen (ZT3) and 15835 bp for the China specimen (ZT1), with similar gene order comprising 37 genes (13 protein-coding genes—PCGs, 2 rRNA genes, and 22 tRNA genes) and a non-coding A + T-rich control region (D-loop) (Table 2, Fig 1, S1 and S2 Tables). The control region was flanked by *rrnS* and *trnI* genes respectively, with 745 bp in *Z*. *tau* ZT3 and 946 bp in *Z*. *tau* ZT1. It contained a long polyT-stretch of 14 bp in *Z*. *tau* ZT3 and 19 bp in *Z*. *tau* ZT1. It also contained in both taxa a long poly A-stretch (20 bp) after 'ATAGA' motif.

**Table 2:** Gene order and features of mitochondrial genome of *Zeugodacus tau*. NC_027290 (China), ZT1 (China), ZT3 (Malaysia).

Gene	Size (bp)	Size (bp)	Size (bp)	Intergenic sequence (bp)	Start/stop codons
	NC_027290	ZT1	ZT3	NC_027290:ZT1:ZT3	NC_027290:ZT1:ZT3
*trnI*(gat)	66	66	66	-3:-3:-3	
*trnQ*(ttg)	69	69	69	8:8:8	
*trnM*(cat)	69	69	69		
*nad2*	1023	1023	1023	9:9:9	All: ATT/TAA
*trnW*(tca)	68	68	68	-9:-8:-8	
*trnC*(gca)	66	66	63	-1:1:1	
*trnY*(gta)	67	67	67	-2:-2:-2	
*cox1*	1534	1534	1534		All: TCG/T
*trnL2*(taa)	66	66	66	4:4:4	
*cox2*	690	690	690	5:5:5	All: ATG/TAA
*trnK*(ctt)	71	71	71		
*trnD*(gtc)	67	67	67		
*atp8*	162	162	162	-7:-7:-7	All: ATT/TAA
*atp6*	678	678	678	-1:-1:-1	All: ATG/TAA
*cox3*	789	789	789	6:6:6	All: ATG/TAA
*trnG*(tcc)	65	65	65	-3:-3:-3	
*nad3*	357	357	357	4:4:4	All: ATA/TAA
*trnA*(tgc)	66	66	4	4:4:4	
*trnR*(tcg)	64	64	64	34:34:34	
*trnN*(gtt)	65	65	65		
*trnS1*(gct)	68	68	68		
*trnE*(ttc)	68	68	68	18:18:18	
*trnF*(gaa)	66	66	66		
*nad5*	1720	1720	1720	15:15:15	All: ATT/T
*trnH*(gtg)	65	65	65	3:3:3	
*nad4*	1341	1341	1341	-7:-7:-7	All: ATG/TAA
*nad4l*	297	297	297	2:2:2	All: ATG/TAA
*trnT*(tgt)	65	65	65		
*trnP*(tgg)	66	66	66	2:2:2	
*nad6*	525	525	525	-1:-1:-1	All: ATT/TAA
*cob*	1137	1137	1137	-2:-2:-2	All: ATG/TAG
*trnS2*(tga)	67	67	67	-65:15:15	
*nad1*	1020	940	940	10:10:10	ATA/TAA:ATA/T:ATA/T
*trnL1*(tag)	65	65	65		
*rrnL*	1327	1327	1327		
*trnV(*tac)	72	72	72		
*rrnS*	792	792	792		
Control region	801	946	745		
Total size	15687	15835	15631		

There were 16 intergenic regions with spacing sequence and 9 regions with overlaps in both *Z*. *tau* ZT3 and *Z*. *tau* ZT1. The region between *trnR* and *trnN* genes in both taxa was separated by the largest sequence of 34 bp. This sequence had clear stem-loop structures.

*Z*. *tau* ZT3 and *Z*. *tau* ZT1 had identical start/stop codons for the 13 PCGs (Table 2, S1 and S2 Tables). Of the start codons, the commonest was ATG (in 6 PCGs–*cox2*, *atp6*, *cox3*, *nad4*, *nad4l*, *cob*), followed by four ATT (*nad2*, *atp8*, *nad5*, *nad6*), two ATA (*nad3*, *nad1*) and one TCG (*cox1*). Nine PCGs had a TAA stop codon (*nad2*, *cox2*, *atp8*, *atp6*, *cox3*, *nad3*, *nad4*, *nad4l*, *nad6*), one had TAG (*cob*), and three had truncated T stop codon (*cox1*, *nad5*, *nad1*).

The nucleotide compositions of the mitochondrial whole genome, protein-coding genes, rRNA genes and control region of *Z*. *tau* ZT3 and *Z*. *tau* ZT1 are summarized in S3 and S4 Tables. Both were A+T rich as expected for mitochondrial genomes. The A + T content for PCGs was lowest in *cox3* (64.8% for *Z*. *tau* ZT3, and 64.6% for *Z*. *tau* ZT1) and highest in *nad4l* (79.8% for *Z*. *tau* ZT3, and 80.1% for *Z*. *tau* ZT1). The A + T content of the non-coding control region was 83.5% for *Z*. *tau* ZT3 and 85.0% for *Z*. *tau* ZT1. For the two ribosomal operons, *rrnL* had a higher A + T content than *rrnS* (79.7% vs 74.7% for *Z*. *tau* ZT3, and 79.6% vs 74.6% for *Z*. *tau* ZT1). The GC skew content which included the whole genome, PCGs, rRNA genes and control region in the two taxa was negative indicating a bias toward the use of Cs over Gs. Although the AT skewness value was positive for the whole genome, rRNA genes and control region, it was variable in the individual PCGs.

As in other insects, the mitogenomes of *Z*. *tau* ZT3 and *Z*. *tau* ZT1 had three main tRNA clusters: (1) I-Q-M; (2) W-C-Y; and (3) A-R-N-S1–E–F (Fig 1). The cloverleaf structure for the respective tRNAs was similar in *Z*. *tau* ZT3 and *Z*. *tau* ZT1. The TψC-loop was absent in *trnF* while *trnS1* lacked the DHU-loop (S1 and S2 Figs).

### Phylogenetic relationship and genetic divergence

**Fig 2:**
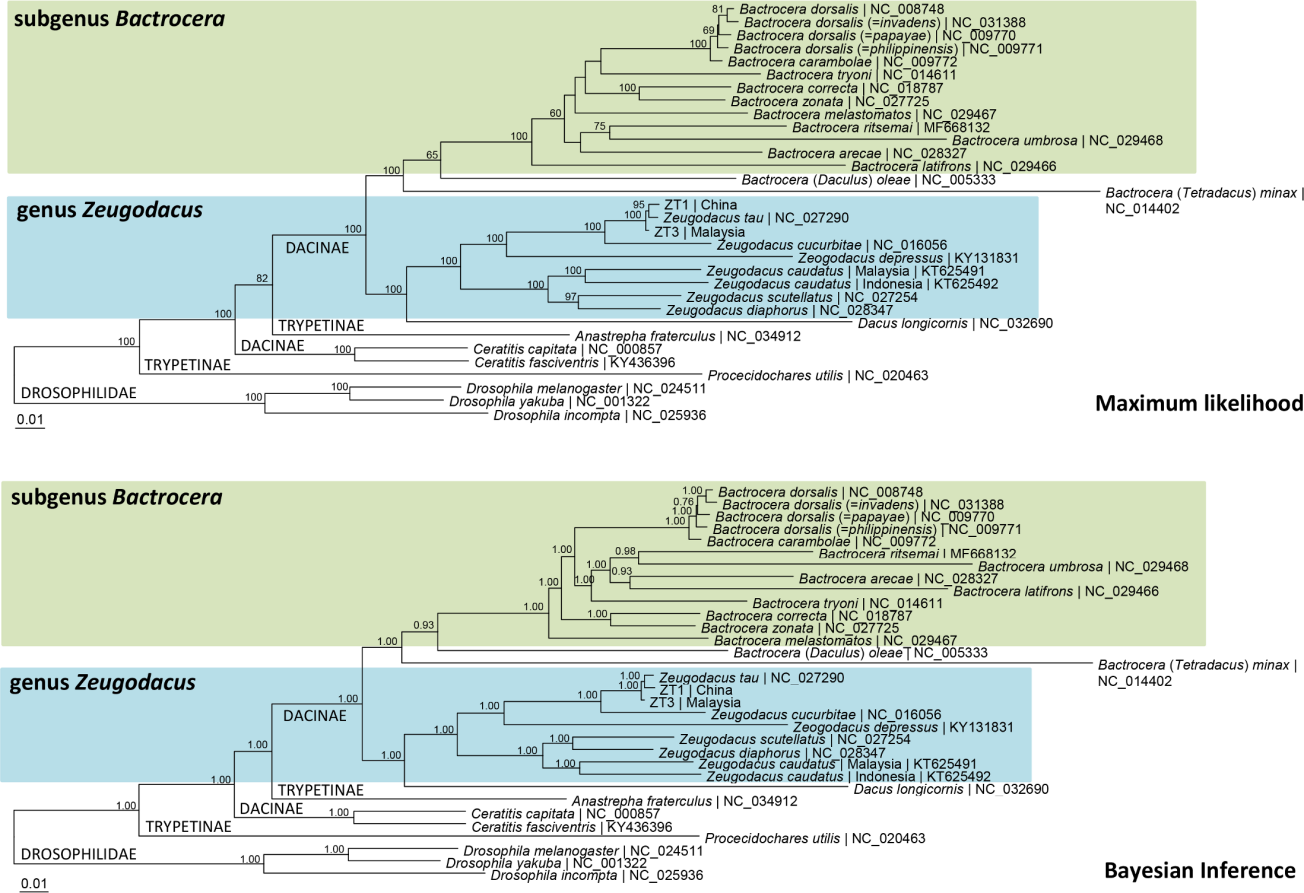
Maximum likelihood and Bayesian inference tree based on 15 mt-genes (13 PCGs and 2 rRNA genes) of the whole mitogenome of *Zeugodacus tau* and other Tephritid fruit flies with Drosophilidae as outgroup. Numeric values at the nodes are ML bootstrap or Bayesian posterior probabilities. The total nucleotide sequences of 15 mt-genes was 13,377 bp with AIC model = GTR+Gamma and BIC model = SYM+Gamma.

Fig 2 depicts the molecular phylogeny of *Z*. *tau* in relation to other congeners and other taxa of the Tephritidae based on 15 mt-genes (13 PCGs + 2 rRNA genes). The phylogram based on 13 PCGs was congruent with that based on 15 mt-genes. Most of the nodes were well-supported. *Z*. *tau* NC_027290 (China) and *Z*. *tau* ZT1 (China) formed a sister group in the lineage containing also *Z*. *tau* ZT3 (Malaysia). The genus *Zeugodacus* was monophyletic and formed a clade with *Dacus longicornis*.

The phylogenetic relationship of some of the component taxa of genus *Bactrocera* was not congruent between ML and BI analyses (Fig 2). For example, ML analysis indicated *B*. *melastomatos* to be a member of the *B*. *dorsalis* complex, but in BI analysis it was basal to the other taxa of subgenus *Bactrocera*. Nonetheless, the genus *Bactrocera* was monophyletic.

**Fig 3:**
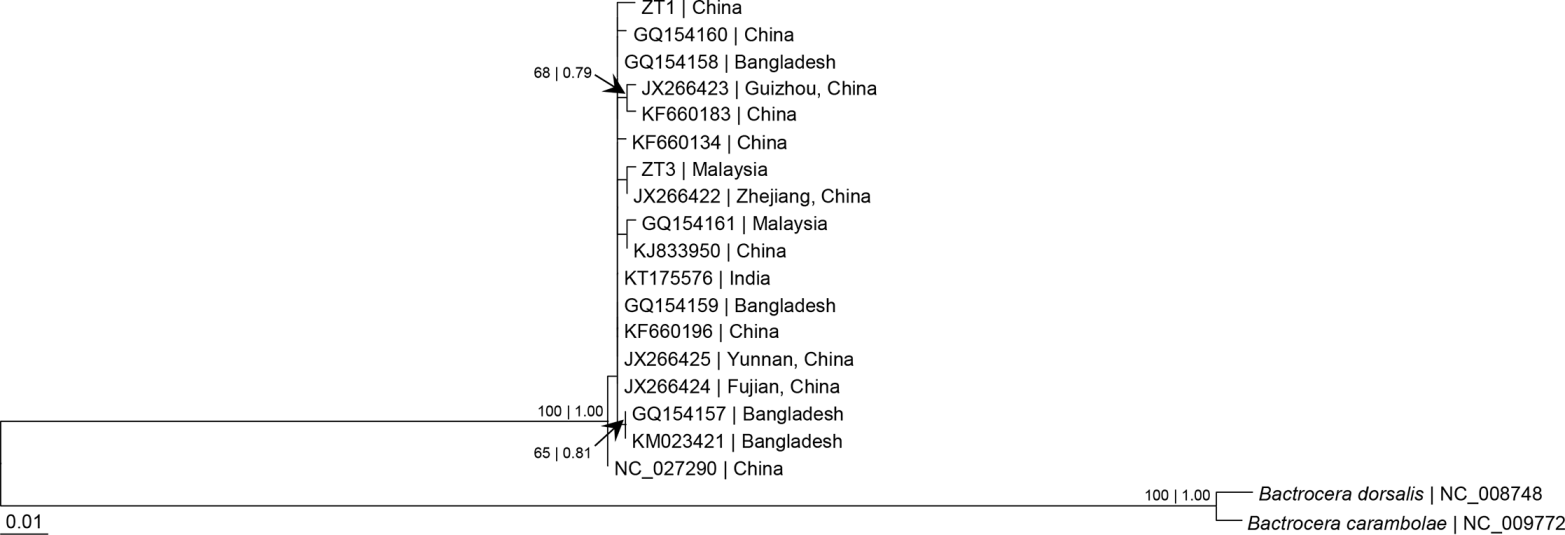
Bayesian inference and maximum likelihood tree based on partial sequence from bp 50–700 of mitochondrial *cox1* gene of *Zeugodacus tau* with *Bactrocera dorsalis* and *B*. *carambolae* as outgroup. Numeric values at the nodes are Bayesian posterior probabilities/ML bootstrap.

Phylogenetic analysis based on partial *cox1* sequences from bp 50–700 indicated that the Z. *tau* taxa from China, Bangladesh, India (Meghalaya, north of Bangladesh) and Malaysia formed a clade with several haplotypes (Fig 3). The uncorrected genetic distance ranged from 'p' = 0 to 'p' = 0.72% (S5 Table).

**Fig 4:**
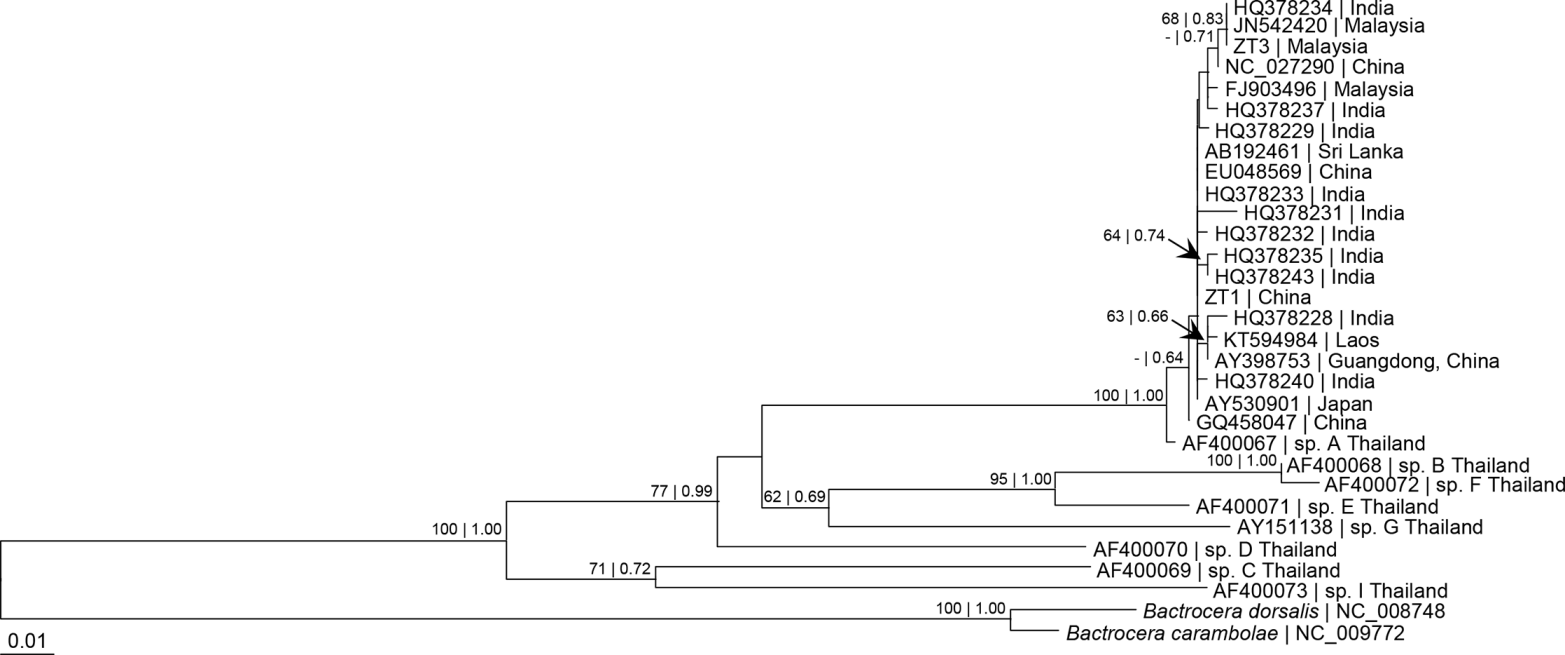
Bayesian inference and maximum likelihood tree based on partial sequence from bp 900–1500 of mitochondrial *cox1* gene of *Zeugodacus tau* with *Bactrocera dorsalis* and *B*. *carambolae* as outgroup. Numeric values at the nodes are Bayesian posterior probabilities/ML bootstrap.

Based on the partial *cox1* sequence from bp 900–1500, the *Z*. *tau* taxa from India, Sri Lanka, Malaysia, Laos, China and Japan formed a clade with *Z*. *tau* sp. A from Thailand (Fig 4), with uncorrected genetic distance ranging from 'p' = 0% to 'p' = 1.39% (S6 Table). This clade was distinctly different from *Z*. *tau* sp. B, C, D, E, F, G, and I from Thailand, with uncorrected genetic distance ranging from 'p' = 9.03% to 'p' = 14.06% (S6 Table).

### Haplotype diversity and nucleotide diversity

**Fig 5:**
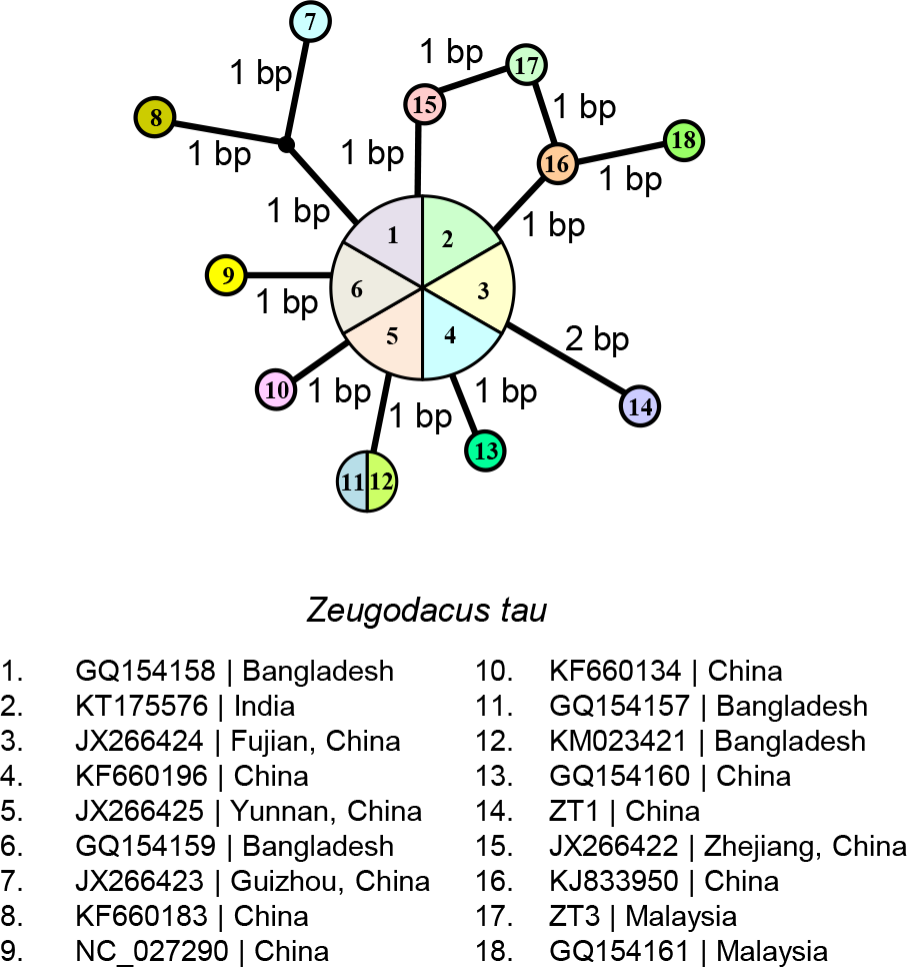
Haplotype network of *Zeugodacus tau* based on cytochrome *c* oxidase subunit I (*cox1*) sequences (from bp 50–700) generated by NETWORK software. Circles represent haplotypes and numbers within the circle represent individuals sharing the specific haplotype.

Twelve haplotypes were revealed in the present 18 *cox1* sequences (from bp 50–700) of *Z*. *tau* from four geographical regions (China, Malaysia, Bangladesh and India) (Fig 5). A common haplotype was found in China (3 sequences), Bangladesh (2 sequences) and India (1 sequence). The haplotype/gene diversity was 0.8954 ± 0.0653, and the nucleotide diversity was 0.0033 ± 0.0022.

**Fig 6:**
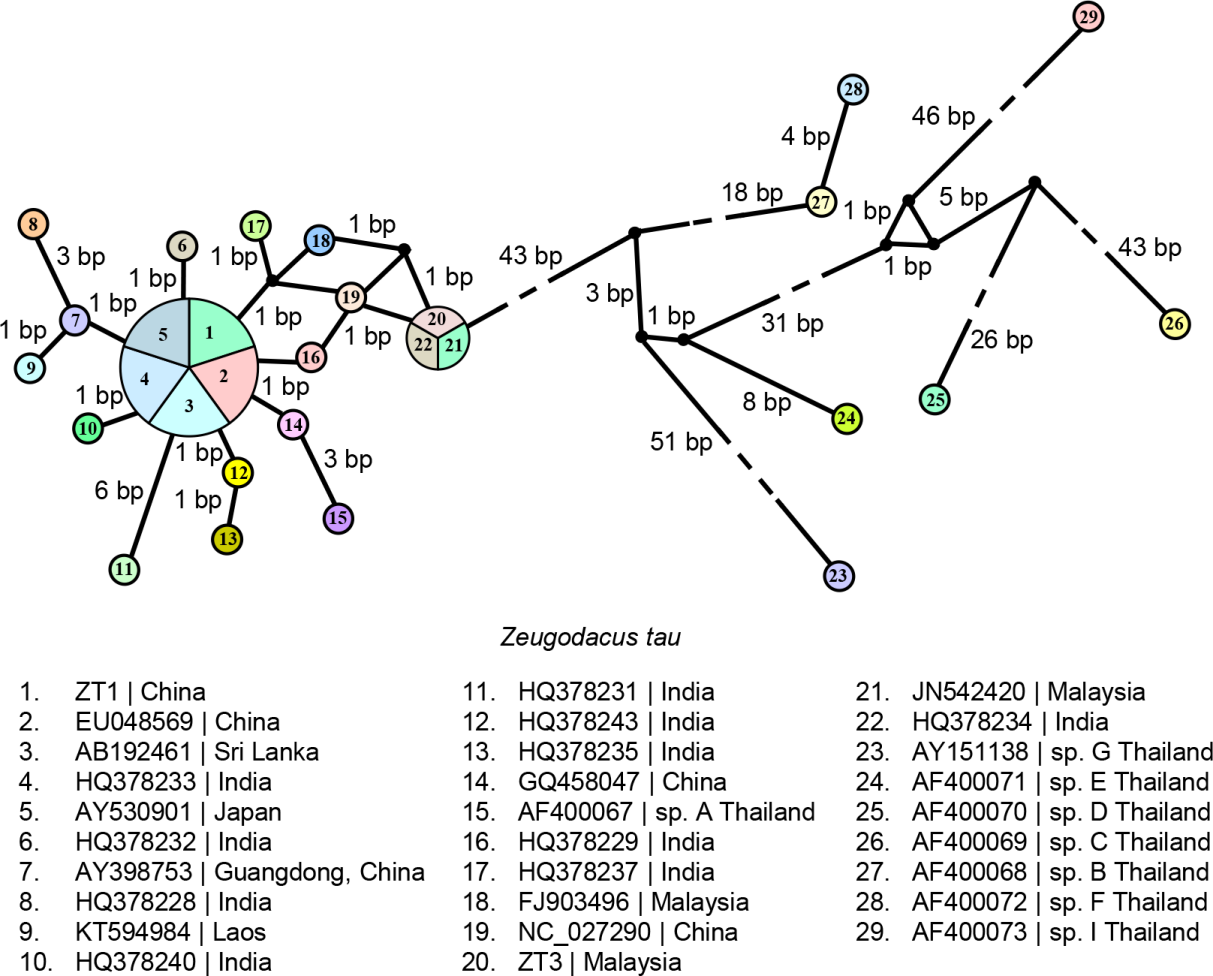
Haplotype network of *Zeugodacus tau* based on cytochrome *c* oxidase subunit I (*cox1*) sequences (from bp 900–1500) generated by NETWORK software. Circles represent haplotypes and numbers within the circle represent individuals sharing the specific haplotype.

Sixteen haplotypes were revealed in the 22 *cox1* sequences (from bp 900–1500) of *Z*. *tau* sensu stricto from six geographical regions (China, Laos, Malaysia, India, Sri Lanka, and Thailand sp. A) (Fig 6). A common haplotype was found in China (2 sequences), Japan (1 sequence), India (1 sequence) and Sri Lanka (1 sequence). Another haplotype was common to Malaysia (2 sequences) and India (1 sequence). The haplotype/gene diversity was 0.9437 ± 0.0372, and the nucleotide diversity was 0.0056 ± 0.0034. *Z*. *tau* sp. B, C, D, E, F, G and I from Thailand formed a distinct cluster from *Z*. *tau* sensu stricto, and each was represented by a distinct haplotype. The haplotypes of *Z*. *tau* F and *Z*. *tau* B had a small difference of 4 bp.

## Discussion

The genus *Zeugodacus* is represented by 52 named and some 19 unnamed species [31]. To date, the complete mitogenome has been reported for seven taxa–*Z*. *caudatus* Malaysia, *Z*. *caudatus* Indonesia, *Z*. *cucurbitae*, *Z*. *depressus*, *Z*. *diaphorus*, *Z*. *scutellatus* and *Z*. *tau* (China). Molecular studies indicate that *Z*. *caudatus* Malaysia and *Z*. *caudatus* Indonesia are sibling species [24,32], and *Z*. *tau* in Thailand consists of eight species [8–10].

The gene order of *Z*. *tau* mitogenome conforms to other *Zeugodacus* and other tephritid mitogenomes [15,20,24,25,28–30]. The mitogenome of *Z*. *tau* ZT3 (Malaysia) is shorter than that of *Z*. *tau* ZT1 (China) and *Z*. *tau* NC_027290 (China), while *Z*. *tau* ZT1 is longer than *Z*. *tau* NC_027290 (Table 2). The difference in the total size of the mitogenome is due mainly to the length of the control region– 745 bp for *Z*. *tau* ZT3, 946 bp for *Z*. *tau* ZT1 and 801 bp for *Z*. *tau* NC_027290 (Table 2).

There are differences in the spacing/overlap sequence in some intergenic regions among *Z*. *tau* mitogenomes: -1 bp in *Z*. *tau* NC_027290 between *trnC* and *trnY* versus 1 bp in *Z*. *tau* ZT3 and *Z*. *tau* ZT1 (Table 2).

The difference in size of the *nad1* gene (1020 bp in *Z*. *tau* NC_027290, and 940 bp in *Z*. *tau* ZT1 and *Z*. *tau* ZT3) and the stop codon (TAA in NC_027290 and incomplete T in ZT1 and ZT3) can be attributed to annotation of the intergenic space between *trnS2* and *nad1* genes (overlap of 65 bp in NC_027290 and spacing sequence of 15 bp in ZT3 and ZT1); this intergenic space is 15 bp in most of the *Zeugodacus* taxa. Incomplete stop codons have been reported in other taxa of tephritid fruit flies [15,20,24]. The incomplete stop codons can be converted to TAA by post-translational polyadenylation [33].

A long poly-A stretch of 20 bp is present in the control region after 'ATAGA' motif in the Malaysian and China taxa of *Z*. *tau*. In addition, a long poly-T stretch is present in the control region of *Z*. *tau* ZT3 (14 bp) and *Z*. *tau* ZT1 (19 bp); this poly-T stretch is not present in *Z*. *tau* NC_027290.

In both *T*. *tau* ZT3 and *Z*. *tau* ZT1, the TΨC-loop was absent in *trnF* while *trnS1* lacked the DHU-loop (S1 and S2 Figs). The TΨC-loop and DHU-loop of tRNA act as special recognition site during protein biosynthesis or translation [34–36]. It has been reported that misacylation of tRNA can affect the survivability of an organism [36]. However, deviant tRNA secondary structures are frequent in Arthropoda [37].

The mitochondrial *cox1* gene has been commonly used for differentiation of various taxa of *Z*. *tau* [9,12,38–46]. In the present study based on partial sequences of *cox1* gene (Figs 3–6), the *Z*. *tau* taxa showed several haplotypes. The taxa from China, Japan, Laos, Malaysia, Bangladesh, Inida, and Sri Lanka were genetically similar to *Z*. *tau* sp. A from Thailand, with 'p' = 0–1.39% (S5 and S6 Tables). As Fuzhou (Foochow), Fujian, China is the type locality of *Z*. *tau*, the taxa from various geographical regions that grouped with those from China can be designated as *Z*. *tau* sensu stricto. Although many taxa had been included for comparison, none were similar to any of the *Z*. *tau* sp. B, C, D, E, F, G, and I reported from Thailand. Among the *Z*. *tau* taxa from Thailand, the genetic distance bewteen *Z*. *tau* F and *Z*. *tau* B was 'p' = 0.69% (S6 Table) with haplotype difference of 4 bp (Fig 6), indicating that these two taxa may be conspecific.

In the present study based on partial *cox1* sequences, *Z*. *tau* ZT1 (China) and *Z*. *tau* NC_027290 (China) were not closely related to each other compared to *Z*. *tau* ZT3 (Malaysia) (Figs 3 and 4). This differs from their closer relationship based on complete *cox1* gene (S3 Fig) and 15 mt-genes (Fig 2). A complete *cox1* gene (or 13 PCGs or 15 mt-genes) instead of partial sequence is therefore more appropriate for determining phylogenetic relationship.

At the higher-level phylogeny, the phylogenetic analysis indicated that *Anastrepha fraterculus* (Tribe Toxotrypanini) of subfamily Trypetinae was grouped with Tribe Dacini of Dacinae, while *Procecidochares utilis* (Tribe Ceceidocharini) of subfamily Trypetinae was basal to the clade containing Dacinae and *A*. *fraterculus* (Fig 2). This discrepancy may be due to insufficient taxon sampling. A broader taxa sampling, particularly Trypetinae, is needed to better elucidate the higher-level phylogeny of the tribes and subfamilies of Tephritidae.

In summary, we have successfully sequenced the complete mitogenome of *Z*.*tau* from Malaysia and China and confirmed that they were conspecific. Based on partial *cox1* sequences, the taxa from China, Japan, Laos, Malaysia, Bangladesh, India, Sri Lanka, and *Z*. *tau* sp. A from Thailand are conspecific and belong to *Z*. *tau* sensu stricto. The mitogenome will prove useful for studies on phylogenetics and systematics of fruit flies of the *Z*. *tau* species complex and other taxa of Tephritidae.

## Supporting information

S1 Fig Cloverleaf structure of the 22 inferred tRNAs in the mitogenome of *Zeugodacus tau* ZT3 (Malaysia). The cloverleaf structure for *trnF* lacked the TψC-loop, and *trnS1* lacked the DHU-loop.
(TIF)

S2 Fig Cloverleaf structure of the 22 inferred tRNAs in the mitogenome of *Zeugodacus tau* ZT1 (China). The cloverleaf structure for *trnF* lacked the TψC-loop, and *trnS1* lacked the DHU-loop.
(TIF)

S3 Fig Bayesian inference and maximum likelihood tree based on complete sequence of mitochondrial *cox1* gene of *Zeugodacus tau* with *Bactrocera dorsalis* and *B*. *carambolae* as outgroup. Numeric values at the nodes are Bayesian posterior probabilities/ML bootstrap.
(TIF)

S1 Table Characteristics of the mitochondrial genome of *Zeugodacus tau* ZT3 (Malaysia). The anticodon of each tRNAs is shown in bracket. J (+) or N (-) indicates gene directions.
(DOCX)

S2 Table Characteristics of the mitochondrial genome of *Zeugodacus tau* ZT1 (China). The anticodon of each tRNAs is shown in bracket. J (+) or N (-) indicates gene directions.
(DOCX)

S3 Table Nucleotide composition of whole mitogenome, protein-coding genes, rRNA genes and control region of *Zeugodacus tau* ZT3 (Malaysia). (DOCX)

S4 Table Nucleotide composition of whole mitogenome, protein-coding genes, rRNA genes and control region of *Zeugodacus tau* ZT1 (China). (DOCX)

S5 Table Uncorrected genetic distance (%) between pairs of *Zeugodacus tau* taxa with *Bactrocera dorsalis* and *B*. *carambolae* as outgroup taxa based on partial sequence from bp 50–700 of mitochondrial *cox1* gene. (DOCX)

S6 Table Uncorrected genetic distance (%) between pairs of *Zeugodacus tau* taxa with *Bactrocera dorsalis* and *B*. *carambolae* as outgroup taxa based on partial sequence from bp 900–1500 of mitochondrial *cox1* gene. (DOCX)
