# Supplementary figures and images for: Complete mitochondrial genome of Zeugodacus tau (Insecta: Tephritidae) and differentiation of Z. tau species complex by mitochondrial cytochrome c oxidase subunit I gene
Source: PLoS One. 2017 Dec 7;12(12):e0189325. doi: 10.1371/journal.pone.0189325 (PMC5720772; doi:10.1371/journal.pone.0189325)

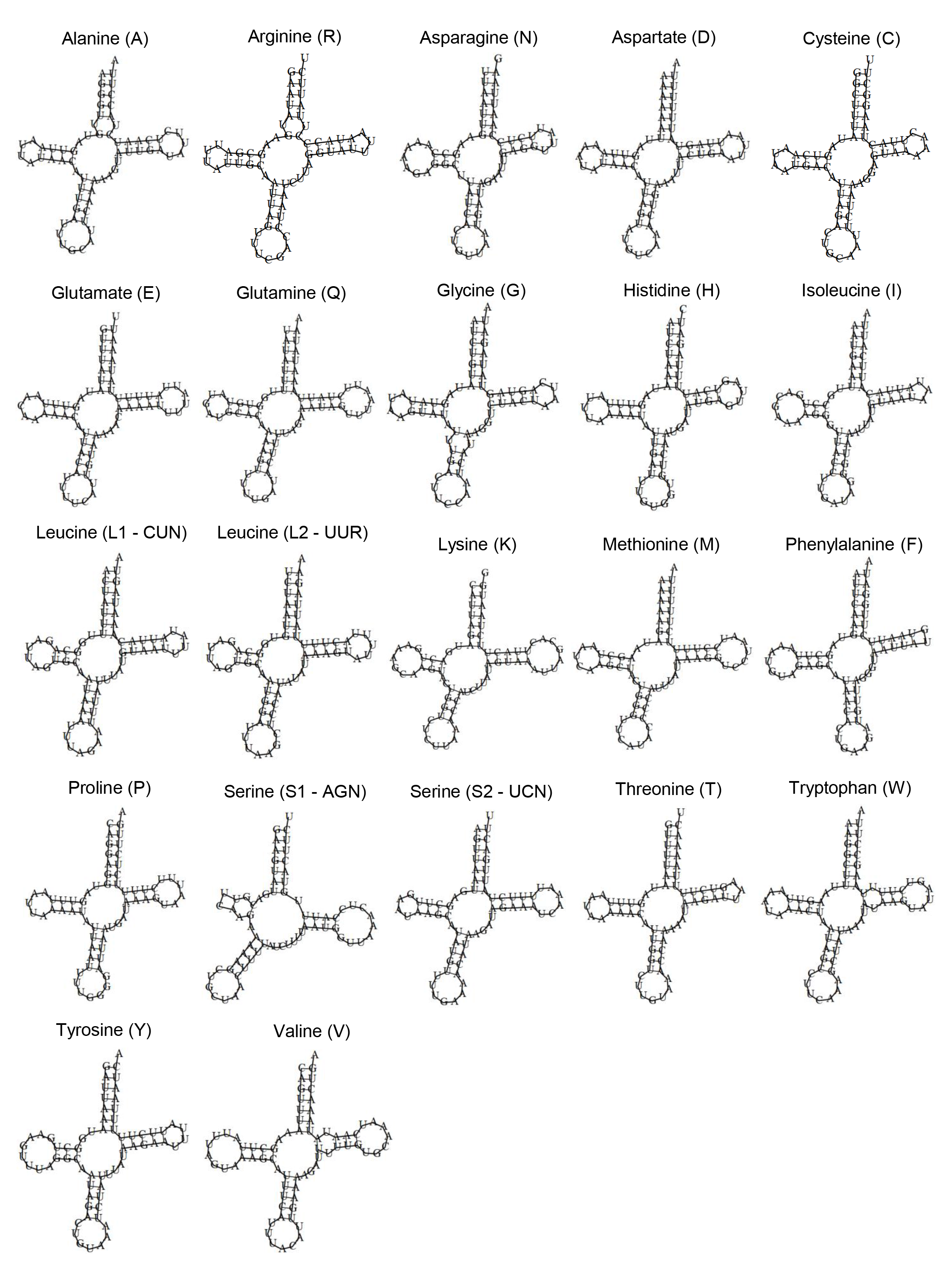

Supplement: S1 Fig — The cloverleaf structure for trnF lacked the TψC-loop, and trnS1 lacked the DHU-loop. (TIF) [file pone.0189325.s001.tif]

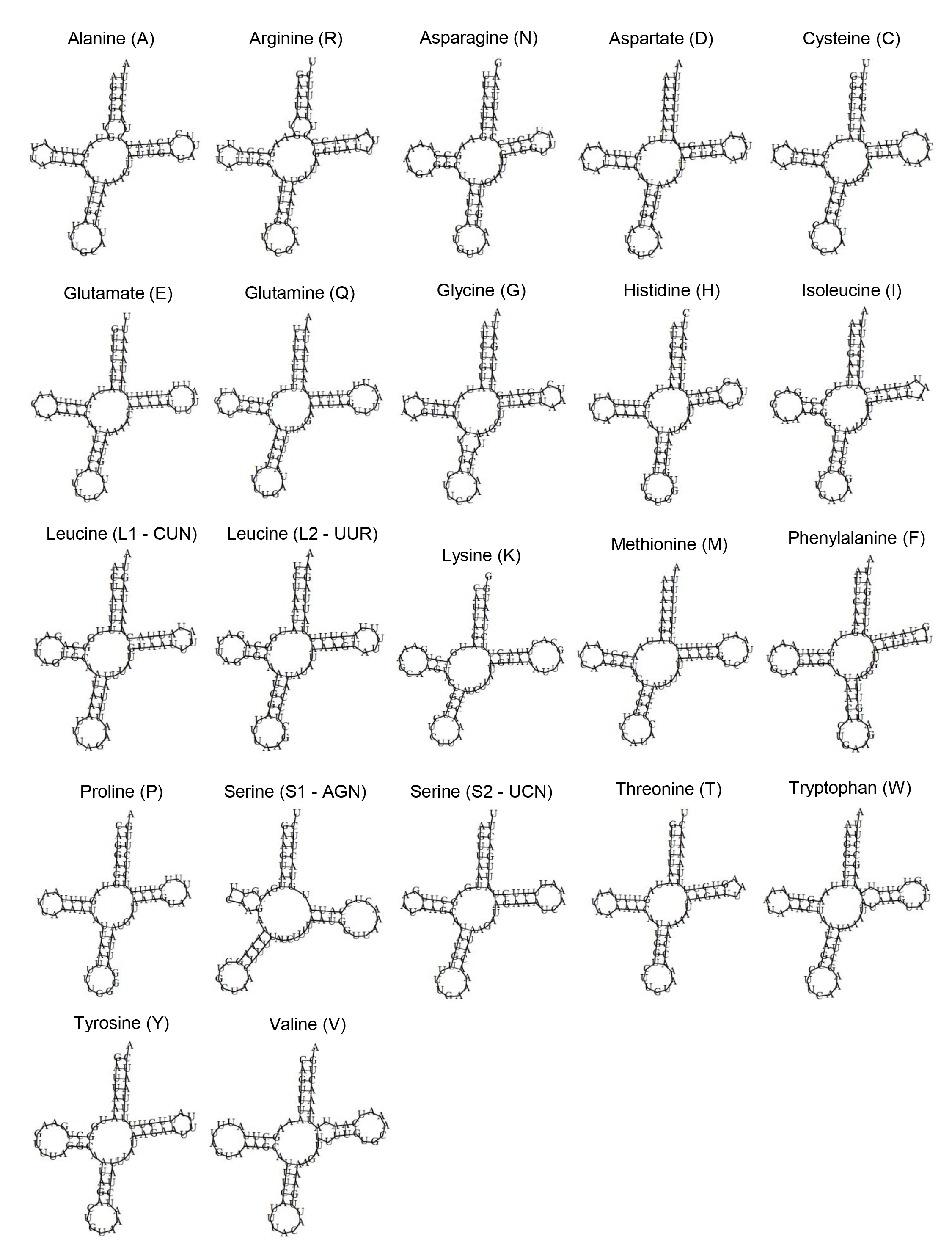

Supplement: S2 Fig — The cloverleaf structure for trnF lacked the TψC-loop, and trnS1 lacked the DHU-loop. (TIF) [file pone.0189325.s002.tif]

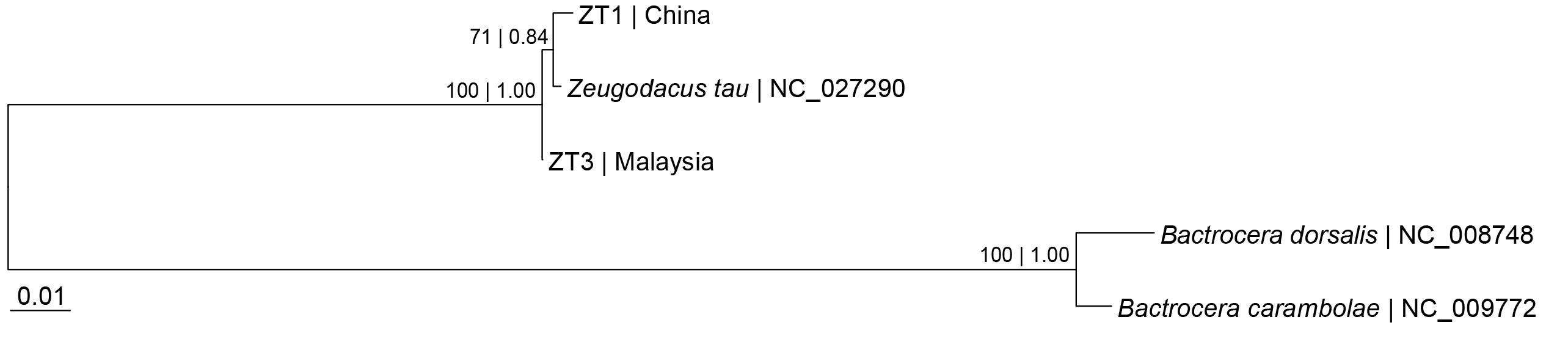

Supplement: S3 Fig — Numeric values at the nodes are Bayesian posterior probabilities/ML bootstrap. (TIF) [file pone.0189325.s003.tif]
