# Supplementary material for: Complete mitochondrial genome of Zeugodacus tau (Insecta: Tephritidae) and differentiation of Z. tau species complex by mitochondrial cytochrome c oxidase subunit I gene
Source: PLoS One. 2017 Dec 7;12(12):e0189325. doi: 10.1371/journal.pone.0189325 (PMC5720772; doi:10.1371/journal.pone.0189325)
Supplement: S1 Table — The anticodon of each tRNAs is shown in bracket. J (+) or N (-) indicates gene directions. (DOCX) [file pone.0189325.s004.docx]

**S1 Table. Characteristics of the mitochondrial genome of *Zeugodacus tau* ZT3 (Malaysia).** The anticodon of each tRNAs is shown in bracket. J (+) or N (-) indicates gene directions.

| Gene | Location | Strand | Size (bp) | Intergenic Sequence | Start/stop codon |
| --- | --- | --- | --- | --- | --- |
| *trnI*(gat) | 1 – 66 | J | 66 | -3 |  |
| *trnQ*(ttg) | 64 – 132 | N | 69 | 8 |  |
| *trnM*(cat) | 141 – 209 | J | 69 |  |  |
| *nad2* | 210 – 1232 | J | 1023 | 9 | ATT/TAA |
| *trnW*(tca) | 1242 – 1309 | J | 68 | -8 |  |
| *trnC*(gca) | 1302 – 1364 | N | 63 | 1 |  |
| *trnY*(gta) | 1366 – 1432 | N | 67 | -2 |  |
| *cox1* | 1431 – 2964 | J | 1534 |  | TCG/T |
| *trnL2*(taa) | 2965 – 3030 | J | 66 | 4 |  |
| *cox2* | 3035 – 3724 | J | 690 | 5 | ATG/TAA |
| *trnK*(ctt) | 3730 – 3800 | J | 71 |  |  |
| *trnD*(gtc) | 3801 – 3867 | J | 67 |  |  |
| *atp8* | 3868 – 4029 | J | 162 | -7 | ATT/TAA |
| *atp6* | 4023 – 4700 | J | 678 | -1 | ATG/TAA |
| *cox3* | 4700 – 5488 | J | 789 | 6 | ATG/TAA |
| *trnG*(tcc) | 5495 – 5559 | J | 65 | -3 |  |
| *nad3* | 5557 – 5913 | J | 357 | 4 | ATA/TAA |
| *trnA*(tgc) | 5918 – 5983 | J | 66 | 4 |  |
| *trnR*(tcg) | 5988 – 6051 | J | 64 | 34 |  |
| *trnN*(gtt) | 6086 – 6150 | J | 65 |  |  |
| *trnS1*(gct) | 6151 – 6218 | J | 68 |  |  |
| *trnE*(ttc) | 6219 – 6286 | J | 68 | 18 |  |
| *trnF*(gaa) | 6305 – 6370 | N | 66 |  |  |
| *nad5* | 6371 – 8090 | N | 1720 | 15 | ATT/T |
| *trnH*(gtg) | 8106 – 8170 | N | 65 | 3 |  |
| *nad4* | 8174 – 9514 | N | 1341 | -7 | ATG/TAA |
| *nad4l* | 9508 – 9804 | N | 297 | 2 | ATG/TAA |
| *trnT*(tgt) | 9807 – 9871 | J | 65 |  |  |
| *trnP*(tgg) | 9872 – 9937 | N | 66 | 2 |  |
| *nad6* | 9940 – 10464 | J | 525 | -1 | ATT/TAA |
| *cob* | 10464 – 11600 | J | 1137 | -2 | ATG/TAG |
| *trnS2*(tga) | 11599 – 11665 | J | 67 | 15 |  |
| *nad1* | 11681 – 12620 | N | 940 | 10 | ATA/T |
| *trnL1*(tag) | 12631 – 12695 | N | 65 |  |  |
| *rrnL* | 12696 – 14022 | N | 1327 |  |  |
| *trnV(*tac) | 14023 – 14094 | N | 72 |  |  |
| *rrnS* | 14095 – 14886 | N | 792 |  |  |
| Control region | 14887 – 15631 | J | 745 |  |  |
