# Supplementary material for: Complete mitochondrial genome of Zeugodacus tau (Insecta: Tephritidae) and differentiation of Z. tau species complex by mitochondrial cytochrome c oxidase subunit I gene
Source: PLoS One. 2017 Dec 7;12(12):e0189325. doi: 10.1371/journal.pone.0189325 (PMC5720772; doi:10.1371/journal.pone.0189325)
Supplement: S2 Table — The anticodon of each tRNAs is shown in bracket. J (+) or N (-) indicates gene directions. (DOCX) [file pone.0189325.s005.docx]

**S2 Table. Characteristics of the mitochondrial genome of *Zeugodacus tau* ZT1 (China).** The anticodon of each tRNAs is shown in bracket. J (+) or N (-) indicates gene directions.

| .Gene | Location | Strand | Size (bp) | Intergenic Sequence | Start/stop codon |
| --- | --- | --- | --- | --- | --- |
| *trnI*(gat) | 1 – 66 | J | 66 | -3 |  |
| *trnQ*(ttg) | 64 – 132 | N | 69 | 8 |  |
| *trnM*(cat) | 141 – 209 | J | 69 |  |  |
| *nad2* | 210 – 1232 | J | 1023 | 9 | ATT/TAA |
| *trnW*(tca) | 1242 – 1309 | J | 68 | -8 |  |
| *trnC*(gca) | 1302 – 1367 | N | 66 | 1 |  |
| *trnY*(gta) | 1369 – 1435 | N | 67 | -2 |  |
| *cox1* | 1434 – 2967 | J | 1534 |  | TCG/T |
| *trnL2*(taa) | 2968 – 3033 | J | 66 | 4 |  |
| *cox2* | 3038 – 3727 | J | 690 | 5 | ATG/TAA |
| *trnK*(ctt) | 3733 – 3803 | J | 71 |  |  |
| *trnD*(gtc) | 3804 – 3870 | J | 67 |  |  |
| *atp8* | 3871 – 4032 | J | 162 | -7 | ATT/TAA |
| *atp6* | 4026 – 4703 | J | 678 | -1 | ATG/TAA |
| *cox3* | 4703 – 5491 | J | 789 | 6 | ATG/TAA |
| *trnG*(tcc) | 5498 – 5562 | J | 65 | -3 |  |
| *nad3* | 5560 – 5916 | J | 357 | 4 | ATA/TAA |
| *trnA*(tgc) | 5921 – 5986 | J | 66 | 4 |  |
| *trnR*(tcg) | 5991 – 6054 | J | 64 | 34 |  |
| *trnN*(gtt) | 6089 – 6153 | J | 65 |  |  |
| *trnS1*(gct) | 6154 – 6221 | J | 68 |  |  |
| *trnE*(ttc) | 6222 – 6289 | J | 68 | 18 |  |
| *trnF*(gaa) | 6308 – 6373 | N | 66 |  |  |
| *nad5* | 6374 – 8093 | N | 1720 | 15 | ATT/T |
| *trnH*(gtg) | 8109 – 8173 | N | 65 | 3 |  |
| *nad4* | 8177 – 9517 | N | 1341 | -7 | ATG/TAA |
| *nad4l* | 9511 – 9807 | N | 297 | 2 | ATG/TAA |
| *trnT*(tgt) | 9810 – 9874 | J | 65 |  |  |
| *trnP*(tgg) | 9875 – 9940 | N | 66 | 2 |  |
| *nad6* | 9943 – 10467 | J | 525 | -1 | ATT/TAA |
| *cob* | 10467 – 11603 | J | 1137 | -2 | ATG/TAG |
| *trnS2*(tga) | 11602 – 11668 | J | 67 | 15 |  |
| *nad1* | 11684 – 12623 | N | 940 | 10 | ATA/T |
| *trnL1*(tag) | 12634 – 12698 | N | 65 |  |  |
| *rrnL* | 12699 – 14025 | N | 1327 |  |  |
| *trnV(*tac) | 14026 – 14097 | N | 72 |  |  |
| *rrnS* | 14098 – 14889 | N | 792 |  |  |
| Control region | 14690 – 15835 | J | 946 |  |  |
