# Supplementary material for: Complete mitochondrial genome of Zeugodacus tau (Insecta: Tephritidae) and differentiation of Z. tau species complex by mitochondrial cytochrome c oxidase subunit I gene
Source: PLoS One. 2017 Dec 7;12(12):e0189325. doi: 10.1371/journal.pone.0189325 (PMC5720772; doi:10.1371/journal.pone.0189325)
Supplement: S3 Table — (DOCX) [file pone.0189325.s006.docx]

**S3 Table. Nucleotide composition of whole mitogenome, protein-coding genes, rRNA genes and control region of *Zeugodacus tau* ZT3 (Malaysia).**

| Region | A/% | C/% | G/% | T/% | A+T/% | G+C/% | AT skew | GC skew |
| --- | --- | --- | --- | --- | --- | --- | --- | --- |
| Whole mitogenome | 38.7 | 16.3 | 10.4 | 34.6 | 73.3 | 26.7 | 0.056 | -0.221 |
| *nad2* | 33.6 | 16.9 | 9.4 | 40.1 | 73.7 | 26.3 | -0.088 | -0.285 |
| *cox1* | 29.6 | 17.4 | 15.9 | 37.1 | 66.7 | 33.3 | -0.112 | -0.045 |
| *cox2* | 32.5 | 18.1 | 13.5 | 35.9 | 68.4 | 31.6 | -0.050 | -0.146 |
| *atp8* | 34.0 | 19.1 | 7.4 | 39.5 | 73.5 | 26.5 | -0.075 | -0.442 |
| *atp6* | 30.8 | 18.7 | 11.8 | 38.7 | 69.5 | 30.5 | -0.114 | -0.226 |
| *cox3* | 29.4 | 20.5 | 14.7 | 35.4 | 64.8 | 35.2 | -0.093 | -0.165 |
| *nad3* | 33.3 | 17.9 | 9.5 | 39.2 | 72.5 | 27.5 | -0.081 | -0.305 |
| *nad5* | 46.1 | 17.0 | 9.2 | 27.7 | 73.8 | 26.2 | 0.249 | -0.298 |
| *nad4* | 47.5 | 16.3 | 8.5 | 27.7 | 75.2 | 24.8 | 0.263 | -0.315 |
| *nad4l* | 51.9 | 13.1 | 7.1 | 27.9 | 79.8 | 20.2 | 0.301 | -0.297 |
| *nad6* | 36.6 | 16.4 | 7.2 | 39.8 | 76.4 | 23.6 | -0.042 | -0.390 |
| *cob* | 30.6 | 18.9 | 13.2 | 37.3 | 67.9 | 32.1 | -0.099 | -0.178 |
| *nad1* | 50.0 | 17.1 | 8.5 | 24.4 | 74.4 | 25.6 | 0.344 | -0.336 |
| *rrnS* | 39.9 | 16.3 | 9.0 | 34.8 | 74.7 | 25.3 | 0.068 | -0.289 |
| *rrnL* | 42.9 | 13.3 | 7.0 | 36.8 | 79.7 | 20.3 | 0.077 | -0.310 |
| Control region | 45.1 | 9.3 | 7.2 | 38.4 | 83.5 | 16.5 | 0.080 | -0.127 |
