# Supplementary material for: Complete mitochondrial genome of Zeugodacus tau (Insecta: Tephritidae) and differentiation of Z. tau species complex by mitochondrial cytochrome c oxidase subunit I gene
Source: PLoS One. 2017 Dec 7;12(12):e0189325. doi: 10.1371/journal.pone.0189325 (PMC5720772; doi:10.1371/journal.pone.0189325)
Supplement: S4 Table — (DOCX) [file pone.0189325.s007.docx]

**S4 Table. Nucleotide composition of whole mitogenome, protein-coding genes, rRNA genes and control region of *Zeugodacus tau* ZT1 (China).**

| Region | A/% | C/% | G/% | T/% | A+T/% | G+C/% | AT skew | GC skew |
| --- | --- | --- | --- | --- | --- | --- | --- | --- |
| Whole mitogenome | 38.8 | 16.2 | 10.4 | 34.6 | 73.4 | 26.6 | 0.057 | -0.218 |
| *nad2* | 33.6 | 16.9 | 9.4 | 40.1 | 73.7 | 26.3 | -0.088 | -0.285 |
| *cox1* | 29.3 | 17.3 | 16.2 | 37.2 | 66.5 | 33.5 | -0.119 | -0.033 |
| *cox2* | 32.6 | 17.8 | 13.4 | 36.2 | 68.8 | 31.2 | -0.052 | -0.141 |
| *atp8* | 34.5 | 20.4 | 6.8 | 38.3 | 72.8 | 27.2 | -0.052 | -0.500 |
| *atp6* | 30.6 | 18.7 | 12.1 | 38.6 | 69.2 | 30.8 | -0.116 | -0.214 |
| *cox3* | 29.5 | 20.7 | 14.7 | 35.1 | 64.6 | 35.4 | -0.087 | -0.169 |
| *nad3* | 33.3 | 18.0 | 9.5 | 39.2 | 72.5 | 27.5 | -0.081 | -0.309 |
| *nad5* | 45.6 | 17.1 | 9.7 | 27.6 | 73.2 | 26.8 | 0.246 | -0.276 |
| *nad4* | 47.3 | 16.5 | 8.6 | 27.6 | 74.9 | 25.1 | 0.263 | -0.315 |
| *nad4l* | 52.2 | 13.2 | 6.7 | 27.9 | 80.1 | 19.9 | 0.303 | -0.327 |
| *nad6* | 36.8 | 16.6 | 7.0 | 39.6 | 76.4 | 23.6 | -0.037 | -0.407 |
| *cob* | 30.4 | 18.8 | 13.4 | 37.4 | 67.8 | 32.2 | -0.103 | -0.168 |
| *nad1* | 50.0 | 16.9 | 8.5 | 24.6 | 74.6 | 25.4 | 0.340 | -0.331 |
| *rrnS* | 39.9 | 16.4 | 9.0 | 34.7 | 74.6 | 25.4 | 0.070 | -0.291 |
| *rrnL* | 42.7 | 13.4 | 7.0 | 36.9 | 79.6 | 20.4 | 0.073 | -0.314 |
| Control region | 46.5 | 8.6 | 6.4 | 38.5 | 85.0 | 15.0 | 0.094 | -0.147 |
