# Supplementary material for: Complete mitochondrial genome of Zeugodacus tau (Insecta: Tephritidae) and differentiation of Z. tau species complex by mitochondrial cytochrome c oxidase subunit I gene
Source: PLoS One. 2017 Dec 7;12(12):e0189325. doi: 10.1371/journal.pone.0189325 (PMC5720772; doi:10.1371/journal.pone.0189325)
Supplement: S5 Table — (DOCX) [file pone.0189325.s008.docx]

**S5 Table. Uncorrected genetic distance (%) between pairs of *Zeugodacus tau* taxa with *Bactrocera dorsalis* and *B. carambolae* as outgroup taxa based on partial sequence from bp 50-700 of mitochondrial *cox1* gene.**

| Taxon | 1 | 2 | 3 | 4 | 5 | 6 | 7 | 8 | 9 | 10 |
| --- | --- | --- | --- | --- | --- | --- | --- | --- | --- | --- |
| 1. *Z. tau* KJ833950 China | - |  |  |  |  |  |  |  |  |  |
| 2. *Z. tau* ZT3 Malaysia | 0.18 | - |  |  |  |  |  |  |  |  |
| 3. *Z. tau* GQ154158 Bangladesh | 0.18 | 0.36 | - |  |  |  |  |  |  |  |
| 4. *Z. tau* KT175576 India | 0.18 | 0.36 | 0.00 | - |  |  |  |  |  |  |
| 5. *Z. tau* JX266424 Fujian | 0.18 | 0.36 | 0.00 | 0.00 | - |  |  |  |  |  |
| 6. *Z. tau* GQ154157 Bangladesh | 0.36 | 0.54 | 0.18 | 0.18 | 0.18 | - |  |  |  |  |
| 7. *Z. tau* KM023421 Bangladesh | 0.36 | 0.54 | 0.18 | 0.18 | 0.18 | 0.00 | - |  |  |  |
| 8. *Z. tau* GQ154159 Bangladesh | 0.18 | 0.36 | 0.00 | 0.00 | 0.00 | 0.18 | 0.18 | - |  |  |
| 9. *Z. tau* GQ154160 China | 0.36 | 0.54 | 0.18 | 0.18 | 0.18 | 0.36 | 0.36 | 0.18 | - |  |
| 10. *Z. tau* KF660196 China | 0.18 | 0.36 | 0.00 | 0.00 | 0.00 | 0.18 | 0.18 | 0.00 | 0.18 | - |
| 11. *Z. tau* JX266425 Yunnan | 0.18 | 0.36 | 0.00 | 0.00 | 0.00 | 0.18 | 0.18 | 0.00 | 0.18 | 0.00 |
| 12. *Z. tau* KF660134 China | 0.36 | 0.54 | 0.18 | 0.18 | 0.18 | 0.36 | 0.36 | 0.18 | 0.36 | 0.18 |
| 13. *Z. tau* JX266422 Zhejiang | 0.36 | 0.18 | 0.18 | 0.18 | 0.18 | 0.36 | 0.36 | 0.18 | 0.36 | 0.18 |
| 14. *Z. tau* KF660183 China | 0.54 | 0.72 | 0.36 | 0.36 | 0.36 | 0.54 | 0.54 | 0.36 | 0.54 | 0.36 |
| 15. *Z. tau* JX266423 Guizhou | 0.54 | 0.72 | 0.36 | 0.36 | 0.36 | 0.54 | 0.54 | 0.36 | 0.54 | 0.36 |
| 16. *Z. tau* GQ154161 Malaysia | 0.18 | 0.36 | 0.36 | 0.36 | 0.36 | 0.54 | 0.54 | 0.36 | 0.54 | 0.36 |
| 17. *Z. tau* NC_027290 China | 0.36 | 0.54 | 0.18 | 0.18 | 0.18 | 0.36 | 0.36 | 0.18 | 0.36 | 0.18 |
| 18. *Z. tau* ZT1 China | 0.54 | 0.72 | 0.36 | 0.36 | 0.36 | 0.54 | 0.54 | 0.36 | 0.54 | 0.36 |
| 19. *B. dorsalis* NC_008748 | 14.65 | 14.83 | 14.83 | 14.83 | 14.83 | 14.65 | 14.65 | 14.83 | 14.65 | 14.83 |
| 20. *B. carambolae* NC_009772 | 13.74 | 13.92 | 13.92 | 13.92 | 13.92 | 13.74 | 13.74 | 13.92 | 13.74 | 13.92 |

| Taxon | 11 | 12 | 13 | 14 | 15 | 16 | 17 | 18 | 19 | 20 |
| --- | --- | --- | --- | --- | --- | --- | --- | --- | --- | --- |
| 11. *Z. tau* JX266425 Yunnan | - |  |  |  |  |  |  |  |  |  |
| 12. *Z. tau* KF660134 China | 0.18 | - |  |  |  |  |  |  |  |  |
| 13. *Z. tau* JX266422 Zhejiang | 0.18 | 0.36 | - |  |  |  |  |  |  |  |
| 14. *Z. tau* KF660183 China | 0.36 | 0.54 | 0.54 | - |  |  |  |  |  |  |
| 15. *Z. tau* JX266423 Guizhou | 0.36 | 0.54 | 0.54 | 0.36 | - |  |  |  |  |  |
| 16. *Z. tau* GQ154161 Malaysia | 0.36 | 0.54 | 0.54 | 0.72 | 0.72 | - |  |  |  |  |
| 17. *Z. tau* NC_027290 China | 0.18 | 0.36 | 0.36 | 0.54 | 0.54 | 0.54 | - |  |  |  |
| 18. *Z. tau* ZT1 China | 0.36 | 0.54 | 0.54 | 0.72 | 0.72 | 0.72 | 0.54 | - |  |  |
| 19. *B. dorsalis* NC_008748 | 14.83 | 15.01 | 15.01 | 15.01 | 15.01 | 14.65 | 14.65 | 14.65 | - |  |
| 20. *B. carambolae* NC_009772 | 13.92 | 14.10 | 14.10 | 14.10 | 14.10 | 13.74 | 13.74 | 13.74 | 1.27 | - |
