# Supplementary material for: Complete mitochondrial genome of Zeugodacus tau (Insecta: Tephritidae) and differentiation of Z. tau species complex by mitochondrial cytochrome c oxidase subunit I gene
Source: PLoS One. 2017 Dec 7;12(12):e0189325. doi: 10.1371/journal.pone.0189325 (PMC5720772; doi:10.1371/journal.pone.0189325)
Supplement: S6 Table — (DOCX) [file pone.0189325.s009.docx]

**S6 Table. Uncorrected genetic distance (%) between pairs of *Zeugodacus tau* taxa with *Bactrocera dorsalis* and *B. carambolae* as outgroup taxa based on partial sequence from bp 900-1500 of mitochondrial *cox1* gene.**

| Taxon | 1 | 3 | 4 | 5 | 6 | 7 | 8 | 9 | 10 | 11 | 12 | 13 | 14 | 15 |
| --- | --- | --- | --- | --- | --- | --- | --- | --- | --- | --- | --- | --- | --- | --- |
| 1. *Z. tau* ZT1 China | - |  |  |  |  |  |  |  |  |  |  |  |  |  |
| 2. *Z. tau* KT594984 Laos | 0.35 | - |  |  |  |  |  |  |  |  |  |  |  |  |
| 3. *Z. tau* ZT3 Malaysia | 0.52 | 0.87 | - |  |  |  |  |  |  |  |  |  |  |  |
| 4. *Z. tau* NC_027290 China | 0.35 | 0.69 | 0.17 | - |  |  |  |  |  |  |  |  |  |  |
| 5. *Z. tau* EU048569 China | 0.00 | 0.35 | 0.52 | 0.35 | - |  |  |  |  |  |  |  |  |  |
| 6. *Z. tau* JN542420 Malaysia | 0.52 | 0.87 | 0.00 | 0.17 | 0.52 | - |  |  |  |  |  |  |  |  |
| 7. *Z. tau* HQ378235 India | 0.35 | 0.69 | 0.87 | 0.69 | 0.35 | 0.87 | - |  |  |  |  |  |  |  |
| 8. *Z. tau* HQ378243 India | 0.17 | 0.52 | 0.69 | 0.52 | 0.17 | 0.69 | 0.17 | - |  |  |  |  |  |  |
| 9. *Z. tau* FJ903496 Malaysia | 0.35 | 0.69 | 0.35 | 0.35 | 0.35 | 0.35 | 0.69 | 0.52 | - |  |  |  |  |  |
| 10. *Z. tau* HQ378237 India | 0.35 | 0.69 | 0.52 | 0.35 | 0.35 | 0.52 | 0.69 | 0.52 | 0.35 | - |  |  |  |  |
| 11. *Z. tau* HQ378228 India | 0.52 | 0.52 | 1.04 | 0.87 | 0.52 | 1.04 | 0.87 | 0.69 | 0.87 | 0.87 | - |  |  |  |
| 12. *Z. tau* AY398753 Guangdong | 0.17 | 0.17 | 0.69 | 0.52 | 0.17 | 0.69 | 0.52 | 0.35 | 0.52 | 0.52 | 0.35 | - |  |  |
| 13. *Z. tau* HQ378232 India | 0.17 | 0.52 | 0.69 | 0.52 | 0.17 | 0.69 | 0.52 | 0.35 | 0.52 | 0.52 | 0.69 | 0.35 | - |  |
| 14. *Z. tau* AB192461 Sri Lanka | 0.00 | 0.35 | 0.52 | 0.35 | 0.00 | 0.52 | 0.35 | 0.17 | 0.35 | 0.35 | 0.52 | 0.17 | 0.17 | - |
| 15. *Z. tau* HQ378229 India | 0.17 | 0.52 | 0.35 | 0.17 | 0.17 | 0.35 | 0.52 | 0.35 | 0.52 | 0.52 | 0.70 | 0.35 | 0.35 | 0.17 |
| 16. *Z. tau* AY530901 Japan | 0.00 | 0.35 | 0.52 | 0.35 | 0.00 | 0.52 | 0.35 | 0.17 | 0.35 | 0.35 | 0.52 | 0.17 | 0.17 | 0.00 |
| 17. *Z. tau* HQ378233 India | 0.00 | 0.35 | 0.52 | 0.35 | 0.00 | 0.52 | 0.35 | 0.17 | 0.35 | 0.35 | 0.52 | 0.17 | 0.17 | 0.00 |
| 18. *Z. tau* GQ458047 China | 0.17 | 0.52 | 0.69 | 0.52 | 0.17 | 0.69 | 0.52 | 0.35 | 0.52 | 0.52 | 0.70 | 0.35 | 0.35 | 0.17 |
| 19. *Z. tau* HQ378240 India | 0.17 | 0.52 | 0.69 | 0.52 | 0.17 | 0.69 | 0.52 | 0.35 | 0.52 | 0.52 | 0.69 | 0.35 | 0.35 | 0.17 |
| 20. *Z. tau* HQ378231 India | 0.70 | 1.05 | 1.22 | 1.04 | 0.70 | 1.22 | 1.05 | 0.87 | 1.04 | 1.05 | 1.22 | 0.87 | 0.87 | 0.70 |
| 21. *Z. tau* HQ378234 India | 0.52 | 0.87 | 0.00 | 0.17 | 0.52 | 0.00 | 0.87 | 0.69 | 0.35 | 0.52 | 1.04 | 0.69 | 0.69 | 0.52 |
| 22. *Z. tau* A AF400067 Thailand | 0.69 | 1.04 | 1.22 | 1.04 | 0.69 | 1.22 | 1.04 | 0.87 | 1.04 | 1.04 | 1.22 | 0.87 | 0.87 | 0.69 |
| 23. *Z. tau* D AF400070 Thailand | 9.55 | 9.90 | 9.03 | 9.20 | 9.55 | 9.03 | 9.55 | 9.38 | 9.38 | 9.38 | 10.09 | 9.72 | 9.72 | 9.55 |
| 24. *Z. tau* F AF400072 Thailand | 11.28 | 11.28 | 11.28 | 11.11 | 11.28 | 11.28 | 11.28 | 11.11 | 11.28 | 11.28 | 11.13 | 11.11 | 11.46 | 11.28 |
| 25. *Z. tau* B AF400068 Thailand | 10.59 | 10.59 | 10.59 | 10.42 | 10.59 | 10.59 | 10.59 | 10.42 | 10.59 | 10.59 | 10.43 | 10.42 | 10.76 | 10.59 |
| 26. *Z. tau* E AF400071 Thailand | 10.07 | 10.07 | 9.55 | 9.72 | 10.07 | 9.55 | 10.07 | 9.90 | 9.90 | 10.07 | 9.91 | 9.90 | 10.24 | 10.07 |
| 27. *Z. tau* G AY151138 Thailand | 10.47 | 10.82 | 10.12 | 10.12 | 10.47 | 10.12 | 10.47 | 10.30 | 10.12 | 10.47 | 11.01 | 10.64 | 10.29 | 10.47 |
| 28. *Z. tau* C AF400069 Thailand | 13.72 | 13.72 | 13.37 | 13.54 | 13.72 | 13.37 | 13.72 | 13.54 | 13.54 | 13.72 | 13.56 | 13.54 | 13.89 | 13.72 |
| 29. *Z. tau* I AF400073 Thailand | 12.85 | 12.67 | 12.50 | 12.67 | 12.85 | 12.50 | 12.85 | 12.67 | 12.67 | 12.85 | 12.69 | 12.67 | 12.85 | 12.85 |
| 30. *B. dorsalis* NC_008748 | 15.45 | 15.80 | 15.28 | 15.28 | 15.45 | 15.28 | 15.45 | 15.28 | 15.28 | 15.45 | 16.00 | 15.63 | 15.45 | 15.45 |
| 31. *B. carambolae* NC_009772 | 15.10 | 15.45 | 14.93 | 14.93 | 15.10 | 14.93 | 15.10 | 14.93 | 14.93 | 14.93 | 15.65 | 15.28 | 15.10 | 15.10 |

| Taxon | 16 | 17 | 18 | 19 | 20 | 21 | 22 | 23 | 24 | 25 | 26 | 27 | 28 | 29 | 30 |
| --- | --- | --- | --- | --- | --- | --- | --- | --- | --- | --- | --- | --- | --- | --- | --- |
| 16. *Z. tau* AY530901 Japan | - |  |  |  |  |  |  |  |  |  |  |  |  |  |  |
| 17. *Z. tau* HQ378233 India | 0.00 | - |  |  |  |  |  |  |  |  |  |  |  |  |  |
| 18. *Z. tau* GQ458047 China | 0.17 | 0.17 | - |  |  |  |  |  |  |  |  |  |  |  |  |
| 19. *Z. tau* HQ378240 India | 0.17 | 0.17 | 0.35 | - |  |  |  |  |  |  |  |  |  |  |  |
| 20. *Z. tau* HQ378231 India | 0.70 | 0.70 | 0.87 | 0.87 | - |  |  |  |  |  |  |  |  |  |  |
| 21. *Z. tau* HQ378234 India | 0.52 | 0.52 | 0.69 | 0.69 | 1.22 | - |  |  |  |  |  |  |  |  |  |
| 22. *Z. tau* A AF400067 Thailand | 0.69 | 0.69 | 0.52 | 0.87 | 1.39 | 1.22 | - |  |  |  |  |  |  |  |  |
| 23. *Z. tau* D AF400070 Thailand | 9.55 | 9.55 | 9.38 | 9.72 | 9.90 | 9.03 | 9.38 | - |  |  |  |  |  |  |  |
| 24. *Z. tau* F AF400072 Thailand | 11.28 | 11.28 | 11.11 | 11.11 | 11.66 | 11.28 | 11.11 | 11.46 | - |  |  |  |  |  |  |
| 25. *Z. tau* B AF400068 Thailand | 10.59 | 10.59 | 10.42 | 10.42 | 10.96 | 10.59 | 10.42 | 11.46 | 0.69 | - |  |  |  |  |  |
| 26. *Z. tau* E AF400071 Thailand | 10.07 | 10.07 | 9.90 | 9.90 | 10.44 | 9.55 | 9.90 | 9.90 | 5.90 | 5.21 | - |  |  |  |  |
| 27. *Z. tau* G AY151138 Thailand | 10.47 | 10.47 | 10.29 | 10.29 | 10.82 | 10.12 | 10.29 | 9.60 | 10.81 | 10.11 | 9.77 | - |  |  |  |
| 28. *Z. tau* C AF400069 Thailand | 13.72 | 13.72 | 13.54 | 13.89 | 14.06 | 13.37 | 13.54 | 11.98 | 12.50 | 11.81 | 12.33 | 12.73 | - |  |  |
| 29. *Z. tau* I AF400073 Thailand | 12.85 | 12.85 | 12.67 | 13.02 | 13.21 | 12.50 | 13.02 | 13.02 | 16.15 | 15.45 | 14.93 | 13.92 | 12.33 | - |  |
| 30. *B. dorsalis* NC_008748 | 15.45 | 15.45 | 15.28 | 15.28 | 15.81 | 15.28 | 15.28 | 14.58 | 16.15 | 15.80 | 15.97 | 15.00 | 15.28 | 14.93 | - |
| 31. *B. carambolae* NC_009772 | 15.10 | 15.10 | 14.93 | 14.93 | 15.46 | 14.93 | 15.10 | 13.72 | 16.15 | 15.80 | 15.80 | 14.65 | 15.28 | 14.76 | 2.78 |
